# Supplementary material for: The influence of travel time to health facilities on stillbirths: A geospatial case-control analysis of facility-based data in Gombe, Nigeria
Source: PLoS One. 2021 Jan 7;16(1):e0245297. doi: 10.1371/journal.pone.0245297 (PMC7790442; doi:10.1371/journal.pone.0245297)
Supplement: S1 File — (DOCX) [file pone.0245297.s001.docx]

**Department of Obstetrics and Gynaecology**

**Federal Teaching Hospital Gombe. Gombe State, Nigeria**

**HEALTHCARE ACCESSIBILITY AND STILLBIRTH REVIEW**

**Instructions:** Fill in this form for every stillbirth and the selected controls within one week of delivery.

**Participant ID:** |__|__|__|__|

Stillbirth **(Case)** *Yes* |__|

Live birth (**Contro**l) *Yes* |__|

Date of delivery: |__|__|/|__|__|__|/|__|__|__|__|

Date of completing questionnaire: |__|__|/|__|__|__|/|__|__|__|__|

# Section 1: Maternal Social and Demographic information

1. Mother’s Age (years): |__|__|
2. Date of birth: |__|__|/|__|__|__|/|__|__|__|__|
3. Mother’s Level of Education: None |__| Primary |__| Secondary |__| Tertiary |__|
4. Mother’s Ethnicity: |__|__|__|__|__|__|__|__|__|__|
5. Mother’s Occupation: Unemployed |__| Informal Employment |__| Formal Employment |__|
6. Partner’s Occupation: Unemployed |__| Informal Employment |__| Formal Employment |__|
7. Partner’s Level of Education: None |__| Primary |__| Secondary |__| Tertiary |__|

# Section 2: Pregnancy and Obstetric information

1. Parity (number of times she has given birth): |__|__|
2. Booking status: Unbooked |__| Booked elsewhere |__| Booked in this facility |__|
3. If booked (elsewhere or in this facility), number of Antenatal visits: |__|__|

# Section 3: Referral Information and mode of transport to this facility

1. Was this mother referred from another facility? Yes |__| No |__|
2. If yes, from which type of facility: Primary facility |__| Secondary facility |__| Tertiary facility |__| Private facility |__|
3. Mode of transportation on day of delivery: Ambulance |__| Commercial transport |__| Motorcycle |__| Personal or family vehicle |__|

# Section 4: Baby’s Condition

1. Weight of the baby (in grams): |__|__|__|__|
2. Sex: Female |__| Male |__|
3. **For stillborn babies**, condition at Birth: Fresh Stillbirth (Intrapartum) |__| Macerated Stillbirth (Antepartum) |__|

# Section 5: Mother’s address and travel time

1. Mother’s address (please limit to suburbs if within Gombe City and to town-level if mother reside outside Gombe City: |__|__ |__|__|__|__|__|__|__|__|__|__|__|__|
2. Latitude of address from OpenStreetMap: |__|__|__|__|__|__|__|
3. Longitude of address from OpenStreetMap: |__|__|__|__|__|__|__|

**Form Completed by**:

Name: …………………….……………. Sign: ………………………… Date: |__|__|/|__|__|__|/|__|__|__|__|
