# Supplementary material for: The influence of travel time to health facilities on stillbirths: A geospatial case-control analysis of facility-based data in Gombe, Nigeria
Source: PLoS One. 2021 Jan 7;16(1):e0245297. doi: 10.1371/journal.pone.0245297 (PMC7790442; doi:10.1371/journal.pone.0245297)
Supplement: S1 Table — (DOCX) [file pone.0245297.s003.docx]

S1 Table. Landcover, road class and their corresponding speeds for modelling travel times

| Description | Description | Speed in km/h | Mode of transport |
| --- | --- | --- | --- |
| Landcover | Forest | 3 | WALKING |
|  | Savanna | 5 | WALKING |
|  | Wetland | 1 | WALKING |
|  | Steppe | 5 | WALKING |
|  | Agriculture | 5 | WALKING |
|  | Water bodies | 0 | WALKING |
|  | Bare soil | 5 | WALKING |
|  | Settlements | 6 | WALKING |
|  | Irrigated agriculture | 4 | WALKING |
|  | Gallery forest and riparian forest | 2 | WALKING |
|  | Agriculture inn shallows and recession | 5 | WALKING |
|  | Woodland | 3 | WALKING |
|  | Sahelian short grass savanna | 5 | WALKING |
|  | Open mine | 1 | WALKING |
| Road | Primary | 100 | MOTORISED |
|  | Secondary | 50 | MOTORISED |
|  | Tertiary | 30 | MOTORISED |
|  | Track | 10 | CYCLING |
