# Supplementary material for: The influence of travel time to health facilities on stillbirths: A geospatial case-control analysis of facility-based data in Gombe, Nigeria
Source: PLoS One. 2021 Jan 7;16(1):e0245297. doi: 10.1371/journal.pone.0245297 (PMC7790442; doi:10.1371/journal.pone.0245297)
Supplement: Attachment — Submitted filename: Response to Reviewers__PONE-D-20-30030.docx [file pone.0245297.s004.docx]

**Journal requirements**

|  | **Journal requirements** | **Authors’ response** |
| --- | --- | --- |
| 1. | Please ensure that your manuscript meets PLOS ONE's style requirements, including those for file naming. The PLOS ONE style templates can be found [HERE](https://journals.plos.org/plosone/s/file?id=wjVg/PLOSOne_formatting_sample_main_body.pdf) and [HERE](https://journals.plos.org/plosone/s/file?id=ba62/PLOSOne_formatting_sample_title_authors_affiliations.pdf) | Thanks for providing the guidelines. We have revised the manuscript to meet the guidelines. |
| 2. | Please include additional information regarding the survey or questionnaire used in the study and ensure that you have provided sufficient details that others could replicate the analyses. For instance, if you developed a questionnaire as part of this study and it is not under a copyright more restrictive than CC-BY, please include a copy, in both the original language and English, as Supporting Information. Moreover, please include more details on how the questionnaire was pre-tested, and whether it was validated. | We have now included a copy of the data collection tool (questionnaire) as supporting information (S1 File) because we developed the questionnaire ourselves and it is not under any copyright. We indicate in the manuscript that the questionnaire was pretested (Page 6, line 2). |
| 3. | In your Methods section, please provide additional information about the participant recruitment method and the demographic details of your participants. Please ensure you have provided sufficient details to replicate the analyses such as: a) the recruitment date range (month and year), b) a description of any inclusion/exclusion criteria that were applied to participant recruitment, c) a table of relevant demographic details**,** d) a statement as to whether your sample can be considered representative of a larger population, e) a description of how participants were recruited, and f) descriptions of where participants were recruited and where the research took place. | The recruitment data range is included on page 5, lines 25-26 of the manuscript (1^st^ January 2019 to 31^st^ December 2019).  The inclusion criterion was women who experienced stillbirths in our facility between 1^st^ January to 31^st^ December 2019 and who consented to participation in the study (Page 5, line 26-28). We have now included information on the exclusion criteria on page 5, lines 29-30).  We have included a statement as to whether our sample can be considered representative of a larger population (Page 5, line 30 to page 6, line 1-3).  We have provided some description on how participants were recruited (page 6, line 9-14).  Information on where participants were recruited and where the research took place is provided on page 5, line 25). |
| 4. | We suggest you thoroughly copyedit your manuscript for language usage, spelling, and grammar. If you do not know anyone who can help you do this, you may wish to consider employing a professional scientific editing service. | The manuscript has been copyedited by one of the authors (Winfred Dotse-Gborgbortsi) to improve clarity, grammar and paragraphs. |
| 5 | Your ethics statement should only appear in the Methods section of your manuscript. If your ethics statement is written in any section besides the Methods, please move it to the Methods section and delete it from any other section. Please ensure that your ethics statement is included in your manuscript, as the ethics statement entered into the online submission form will not be published alongside your manuscript. | We have now moved the ethics statement to the ethics section of the manuscript (page 8, line 14-17). |
| 6. | We note that Figure 2 in your submission contain map images which may be copyrighted. All PLOS content is published under the Creative Commons Attribution License (CC BY 4.0), which means that the manuscript, images, and Supporting Information files will be freely available online, and any third party is permitted to access, download, copy, distribute, and use these materials in any way, even commercially, with proper attribution. For these reasons, we cannot publish previously copyrighted maps or satellite images created using proprietary data, such as Google software (Google Maps, Street View, and Earth).  We require you to either (a) present written permission from the copyright holder to publish these figures specifically under the CC BY 4.0 license, or (b) remove the figures from your submission | Figures 1, 3 and 4 are maps which were made by the authors. The maps have not been previously published and were produced by the authors with administrative boundaries data (basemaps) from geoBoundaries (<https://journals.plos.org/plosone/article?id=10.1371/journal.pone.0231866>), and OpenStreetMap (<https://www.openstreetmap.org/#map=14/10.2251/11.1767&layers=G>). These basemaps source are online, open license resources of the geographic boundaries of political administrative divisions of countries. |
| 7. | Please include captions for your Supporting Information files at the end of your manuscript, and update any in-text citations to match accordingly. Please see our Supporting Information guidelines for more information: http://journals.plos.org/plosone/s/supporting-information | We have provided the caption for our supplementary table. |

|  | **Comments for the Authors (Reviewer #1)** | **Authors’ response** |
| --- | --- | --- |
| 1. | Wariri et al looked at the influence of travel time to health facilities on stillbirths in Gombe, Nigeria. This is an exciting study in attempt at shedding light on the myriad of factors that contribute to the unacceptably high rate of stillbirths in most African countries. However, they need to address the issues below to improve the quality of the publication. | We thank the editor and the reviewers for the time spent in reviewing our manuscript and for providing detailed comments which we believe have improved the quality of our manuscript.  We have provided a point-by-point response to the reviewers’ comments below.  **Note that all references to page and line numbers are based on the revised manuscript document with tracked changes.** |
| 2. | What is the influence of existing referral strategy on decision making on where to seek care first by mothers? Is there a bypassing dynamic in the study population? What shapes it? | Thank you for raising these important questions which would further contextualise the findings reported in this manuscript. While anecdotal reports exist related to the bypassing of primary and secondary health facilities by mothers to seek care in the only tertiary health facility in Gombe (our study population), to the best of our knowledge, there is no documented peer-reviewed evidence to back this claim. Thus, we are unable to explicitly provide an answer on the influence of the existing referral strategy on the decision making on where to seek care by mothers or what shapes such behaviour.  In an attempt to answer these very important questions, we had tried to explore the reasons behind bypassing the nearest primary or secondary facilities by the mothers in our study population (Page 15, paragraph 2). In Page 15, Lines 19-23, we identified the quality of maternal and newborn services offered public-sector primary and secondary facilities as possible reasons shaping bypassing or decision on where to seek care by mothers. |
| 3. | It would have been nice to show flow of patients to the tertiary hospital in a map to better understand the influence of residence on choice of facility. | Thank you for this brilliant suggestion. We have added Fig 3 which shows the flow patterns of still births only. We excluded the controls (live births) from this new map. |
| 4. | Geographically inaccessibility directly affects the second delay in the three-delay concept by Thaddeus and Maine, rather than the indirect impact it has on the first delay. Discussion on second delay needed as well. | We agree with the reviewer on the point raised here. In fact, in the introduction section, on page , lines 6 – 10, we discuss and imply that geographical inaccessibility indirectly impact the first-delay by creating a lack of interest to seek care, as well as impacting directly the second-delay by increasing the travel time in reaching health facilities. We appropriately referenced our assertions (ref 12 – 14). |
| 5. | Is there a recommended travel time to nearest hospital facilities which reduces occurrence of stillbirths during delivery? | To our knowledge, there is no recommended travel time to nearest health facility which reduces the occurrence of stillbirths. |
| 6 | Include the precise coordinate location of the study setting, inclusion of study area map will be helpful. | Thank you for the suggestion. We have added a map of the study area. We did not add the precise coordinate of the study location but rather shown it on the new map. See Fig 1 on page 5 |
| 7. | Why did you select the travel time model via AccessMod? Justify selection of this method to measure travel time. | Thank you for this comment. We have now justified why we used AccessMod and travel time estimates. A summary of our revision is that, travel time is better than Euclidean and network distance. Also, AccessMod is free, easy to use and widely used for similar purposes. See page 7, lines 2 – 5. |
| 8. | Review AccessMod documentation on how to handle roads and water bodies features in modelling access rather than eliminating them. | We have revised this section to improve clarity. We did not eliminate roads and water bodies. What AccessMod does is to remove the artificial bridge. This is explained in Section 5.5.2 and other parts of the AccessMod user manual. See lines 15 – 18 on page 7. |
| 9. | How did you in-cooperate the changing elevation/slope in walking and cycling speed? Consider using the Tobbler’s equation. | The variations in walking/cycling speed due to changing elevation was implemented using the Tobler’s equation. As we wanted to improve readability for a wider audience including health workers and policy makers with less or without GIS knowledge, we avoided using such technical terms. However, we have revised this section to mention Tobler and direct readers to the user manual and an associated paper (<https://link.springer.com/article/10.1186/1476-072X-7-63>) for more technical readers. See lines 19 – 22 on page 7. |
| 10. | Consider including the odd ratios of the stillbirths and intrapartum stillbirths in Table 1 and Table 2 respectively. | Thank you for this comment. We, however, retain the proportions and percentages as presented in Table 1 and 2 in the manuscript. We retain these rather than the odds ratios as suggested because the tables show the comparison of the frequency of key sociodemographic characteristics by livebirths vs stillbirths (Table 1) and antepartum vs intrapartum stillbirths (Table 2). We were NOT comparing the relative **odds** of the occurrence of the outcome of interest (stillbirths OR antepartum/ intrapartum stillbirths), given exposure to the variable of interest (in this case, the sociodemographic characteristics). Table 4 includes odds ratios because we were comparing the odds of the outcome (stillbirths) given the exposure (travel time to nearest facility and to the FTHG). |
| 11. | Did you identify the referral cases? From which level and type of facilities were they referred from? | We identified the cases that were referred in Table 1 and Table 2. Table shows that 73% (116/159) of stillbirths (cases) were referred compared to 17% (27/159) of live births (controls). Table 2 (page 9) gives the comparison of referral status between antepartum and intrapartum stillbirths.  Both tables also provide information on the level/type of facility (facility referred from) where the cases or cases have been referred from. For example, in Table 1 (page 8), 35% (40/116) of stillbirths were referred from primary healthcare centres, 47.9% (56/116) from secondary healthcare facilities etc. Again, Table 2 (page 9) gives the comparison of which facilities referred from (type/level), for antepartum and intrapartum stillbirths |
| 12. | Review on how to capture the SD, OR and CI ranges correctly within a sentence. Include the quantifying percentages in the sentences rather than at the end. | Thank you for this comment. We have now implemented the suggested edits throughout the result section of the manuscript. |
| 13. | What could be the possible reasons why the majority of women used the Gombe tertiary hospital rather than their nearest facilities within the state, before you compare with other state and prior studies? | Thank you for this comment. We have edited paragraph 2 of the discussion section (page 15) to reflect this comment. In brief, we argue that the poor quality and variability of public primary and secondary facilities within the State could partly explain why most women sought care in the FTH Gombe, rather that their nearest facilities within the state. |
| 14. | State the specific mean travel times of the prior studies you are comparing with, state the localities of those studies and state possible reasons for attaining similar mean travel time in your study | We have provided additional information as requested for the studies we compared our finding with. See page 15, line 24-28. We were unable to compare our mean travel time to the more related study in Africa by Karra et al (38) because their travel times are self-reported and prone to reporting and recall bias. More so, their travel time estimates were presented in grouped categories. |
| 15. | Discuss and put into context why women with stillbirth are poorer, less educated etc in your study setting. Compare with prior similar studies. | Thank you for raising this comment. We have now contextualised why women with stillbirths are poorer and less educated (page 16, lines 1-9). We also compared our finding with prior similar studies from Gombe (our setting), Nepal, and Span (page 16, lines 8-9). |
| 16. | Can the lowest administration unit be used for the purpose of uniformity to represent the residence location of the mother instead of using two (i) areas of residence and, (ii) town of residence? | Thank you for the suggestion. We have generalised location address to town of residence. We reported only town level. We have changed the wording in the methods and clarified that we considered suburbs of the Gombe city itself as towns else the whole Gombe City as one unit/town will be spatially incomparable in size to the other towns. Page 6 lines 15 - 19 |
| 17. | AccessMod5 travel time surface is normally generated on the assumption that the care seeker uses the nearest health facility. It’s one of the limitations of the travel time model. How did you overcome this limitation as stated in your discussion? (Review on the merits and demerits of using a travel time model). | Thank you for reminding us to this inherent AccessMod limitation. Actually, this limitation was a strength in our analysis. First, we modelled travel times to the nearest health facility. Subsequently, we used only the tertiary facility at Gombe as our destination as all the study participants accessed care there. Therefore, we modelled a theoretical/expected/ideal travel time to the nearest health facility and then to the Federal Teaching Hospital Gombe where all study participants sought care. Thus, we avoided the assumption of modelling everyone to the nearest facility similar to studies using the Demographic and Health Survey data due to unknown destinations. We have explained this in our discussion (page 17, lines 17-23), thus, no revisions were implemented. |
| 18. | Discuss specific interventions needed for quality obstetric and newborn care in your recommendation section in view of your findings. | We have expanded this section to include specific recommendations needed for quality obstetric and newborn care (page 17, lines 4-7). |
| 19. | Strengthen the conclusion to summarize the study, major findings and way forward on reducing stillbirth counts in Gombe state. | We have now strengthened the conclusion section of the manuscript as suggested (page 18, lines 5-12). |
